# A Multilingual Digital Mental Health and Well-Being Chatbot (ChatPal): Pre-Post Multicenter Intervention Study

**DOI:** 10.2196/43051

**Published:** 2023-07-06

**Authors:** Courtney Potts, Frida Lindström, Raymond Bond, Maurice Mulvenna, Frederick Booth, Edel Ennis, Karolina Parding, Catrine Kostenius, Thomas Broderick, Kyle Boyd, Anna-Kaisa Vartiainen, Heidi Nieminen, Con Burns, Andrea Bickerdike, Lauri Kuosmanen, Indika Dhanapala, Alex Vakaloudis, Brian Cahill, Marion MacInnes, Martin Malcolm, Siobhan O'Neill

**Affiliations:** 1 School of Psychology Ulster University Coleraine United Kingdom; 2 Department of Social Sciences,Technology and Arts Luleå University of Technology Luleå Sweden; 3 School of Computing, Ulster University Belfast United Kingdom; 4 Department of Accounting, Finance & Economics, Ulster University Belfast United Kingdom; 5 Department of Health, Education and Technology Luleå University of Technology Luleå Sweden; 6 Department of Sport, Leisure & Childhood Studies Munster Technological University Cork Ireland; 7 School of Art Ulster University Belfast United Kingdom; 8 Department of Social and Health Management University of Eastern Finland Kuopio Finland; 9 Department of Nursing Science University of Eastern Finland Kuopio Finland; 10 Nimbus Research Centre Munster Technological University Cork Ireland; 11 Research & Innovation, National Health Service Western Isles Scotland United Kingdom; 12 Public Health Intelligence and Information Services National Health Service Western Isles Scotland United Kingdom

**Keywords:** conversational user interfaces, digital interventions, Warwick-Edinburgh Mental Well-Being Scale, Satisfaction With Life Scale, World Health Organization-Five Well-Being Index Scale, mental health, apps, health care, mixed methods, conversation agent, mental well-being, digital health intervention

## Abstract

**Background:**

In recent years, advances in technology have led to an influx of mental health apps, in particular the development of mental health and well-being chatbots, which have already shown promise in terms of their efficacy, availability, and accessibility. The ChatPal chatbot was developed to promote positive mental well-being among citizens living in rural areas. ChatPal is a multilingual chatbot, available in English, Scottish Gaelic, Swedish, and Finnish, containing psychoeducational content and exercises such as mindfulness and breathing, mood logging, gratitude, and thought diaries.

**Objective:**

The primary objective of this study is to evaluate a multilingual mental health and well-being chatbot (ChatPal) to establish if it has an effect on mental well-being. Secondary objectives include investigating the characteristics of individuals that showed improvements in well-being along with those with worsening well-being and applying thematic analysis to user feedback.

**Methods:**

A pre-post intervention study was conducted where participants were recruited to use the intervention (ChatPal) for a 12-week period. Recruitment took place across 5 regions: Northern Ireland, Scotland, the Republic of Ireland, Sweden, and Finland. Outcome measures included the Short Warwick-Edinburgh Mental Well-Being Scale, the World Health Organization-Five Well-Being Index, and the Satisfaction with Life Scale, which were evaluated at baseline, midpoint, and end point. Written feedback was collected from participants and subjected to qualitative analysis to identify themes.

**Results:**

A total of 348 people were recruited to the study (n=254, 73% female; n=94, 27% male) aged between 18 and 73 (mean 30) years. The well-being scores of participants improved from baseline to midpoint and from baseline to end point; however, improvement in scores was not statistically significant on the Short Warwick-Edinburgh Mental Well-Being Scale (*P*=.42), the World Health Organization-Five Well-Being Index (*P*=.52), or the Satisfaction With Life Scale (*P*=.81). Individuals that had improved well-being scores (n=16) interacted more with the chatbot and were significantly younger compared to those whose well-being declined over the study (*P*=.03). Three themes were identified from user feedback, including “positive experiences,” “mixed or neutral experiences,” and “negative experiences.” Positive experiences included enjoying exercises provided by the chatbot, while most of the mixed, neutral, or negative experiences mentioned liking the chatbot overall, but there were some barriers, such as technical or performance errors, that needed to be overcome.

**Conclusions:**

Marginal improvements in mental well-being were seen in those who used ChatPal, albeit nonsignificant. We propose that the chatbot could be used along with other service offerings to complement different digital or face-to-face services, although further research should be carried out to confirm the effectiveness of this approach. Nonetheless, this paper highlights the need for blended service offerings in mental health care.

## Introduction

On a global scale, the need for mental health–related services has been on the rise [1]. Worryingly, according to Waumans et al [2], many of those who need mental health care do not seek it, or alternatively, encounter barriers that discourage help-seeking. Interestingly, in Western European countries, it appears that lack of mental health literacy, social stigma, waiting lists, and logistical difficulties constitute the predominant barriers to help-seeking behaviors, as opposed to financial or socioeconomic constraints [2]. There is also a need to educate citizens about mental health to minimize stigma and increase confidence in taking appropriate actions [3]. In order to meet the demand for mental health care and educate people about mental health, many research projects have focused on digital solutions, including artificial intelligence (AI)–based services [1,4-7]. Although digital mental health tools have been in development for some time, the critical years of the COVID-19 pandemic have contributed to long-term challenges and changes in how services are delivered [8-11]. However, it is important to note that not all apps that brand themselves as “mental health apps” have been rigorously evaluated and may lack content that has been supported by the scientific literature or have not been created in collaboration with mental health professionals and potential end users [12].

Recent advances in the areas of AI and natural language processing have enabled a rapid increase in the number of mental health and well-being chatbots being developed. Some mental health chatbots that have been trialed include Woebot, Wysa, Shim, Vivibot, and Tess [4,13-16]. Previous studies have used randomized controlled trials [4,14-16], lasting between 2 weeks and 8 weeks, with sample sizes ranging from 45 to 74 participants. Another study used a quasi-experimental “in-the-wild” study design [13], trialing the chatbot “Wysa” for 8 weeks with 129 users. The chatbot known as “Youper” was tested in the wild for acceptability and effectiveness with 4517 users [17].

Researchers found that using a mental health chatbot contributed to higher engagement with the material and a higher increase in emotional awareness compared to using an informational book [4,16]. Wysa, which brands itself as an emotionally intelligent mobile chatbot app, was found to contribute to improved mental well-being among those who used it often [13]. While previous research on mental health chatbots shows mainly positive effects on well-being or higher engagement compared to traditional ways of self-management, conversations with chatbots may still feel robotic [14]. Chatbots such as Shim and Vivibot use prewritten text via a decision tree structure, and both chatbots have shown mild to moderate, statistically significant positive effects on mental well-being during trials [14,15]. While effectiveness is important, acceptability among users is equally important. Youper, for example, scored high both on user retention (over 60% more than 1 week and over 40% after 4 weeks) and acceptability while still showing evidence of being effective [17].

As established by previous research, the need for mental health care has increased, and providers struggle to meet the current needs [1,8,9]. While it is important to increase capacity for treatment, preventative measures should also be considered. Increasing mental health literacy could help combat the rising need for mental health care by educating the public about mental health and when to seek help, and reducing stigma [2,18]. Increasing mental health literacy can help improve help-seeking behavior, meaning that people may seek out help earlier, reducing the impact of the illness [2,19]. Previous research surrounding mental health chatbots shows promising results, meaning that there is potential for conversational chatbots to increase mental health literacy and improve mental well-being among users [4,13].

The ChatPal project [20], funded by the Northern Periphery and Arctic (NPA) Programme, involves the design, development, and trialing of a mental health chatbot to support the well-being of individuals in rural NPA areas [21]. Mental health care professionals were surveyed to establish their views toward health-related chatbots [22], and workshops were held across NPA regions to gather user needs [5]. The ChatPal chatbot was subsequently developed based on use cases that professionals endorse and to meet the needs of end users. The chatbot is a mental health promotion tool available in 4 languages, including English, Scottish Gaelic, Swedish, and Finnish. The chatbot is not designed to diagnose or treat those with severe mental illness but instead helps promote good mental health and well-being. This paper focuses on the evaluation of a pre-post intervention study for the ChatPal chatbot.

The aim of this study is to establish if using a multilingual mental health and well-being chatbot (ChatPal) has an effect on mental well-being. The secondary objectives of the research are to establish the characteristics of those who had improved mental well-being compared to those whose mental well-being declined and to identify themes through qualitative analysis of user feedback.

## Methods

### Overview

The methodology for this study was a single-arm pre-post intervention study, which involved the recruitment of participants to use the ChatPal chatbot for a 12-week period. Participants’ well-being was measured at baseline, after 6 weeks, and at the end of 12 weeks, and feedback was gathered through the chatbot, surveys, and focus groups. This was a mixed methods study that included a quantitative evaluation of pre- and postmental well-being scale scores, an analysis of chatbot usage data, and a qualitative analysis of participant feedback.

### Ethics Approval

This study received ethical approval from each institution involved in the research: the Ulster University Research Ethics Committee (Reference numbers REC.21.0021 and FCPSY-21-038-A), the Munster Technological University Research Ethics Committee (Reference Number MTU21034A), the Ethics Review Authority in Sweden (Reference Etikprövningsmyndigheten number 2020-00808), and the University of Eastern Finland Committee on Research Ethics (Statement 14/2021). All participants gave written informed consent to take part in the study. No form of compensation, monetary or otherwise, was offered to participants. All data collected from participants, including chatbot event log data and surveys, was anonymous.

### Intervention

The ChatPal chatbot was developed using Rasa (backend; Alan Nichol and Alex Weidauer) and PhoneGap (front end; Nitobi), with communication between backends using HTTP requests or responses (Multimedia Appendix 1). Upon receiving user inputs from the ChatPal app, the Rasa stack forward the user input to the Rasa Natural Language Understanding unit, which extracts user intentions (intents) and relevant metadata (entities) from the input. Once the intents and entities are identified, the chatbot’s response to the user is decided by the Rasa core. Rasa supports various responses, including text messages, buttons, images, videos, and reminders, all of which were used in ChatPal. Rasa SDK was used to execute custom actions separately on an action server. All user interactions with ChatPal (user event log data) were stored in the Amazon Relational Database Service.

**Figure 1:**
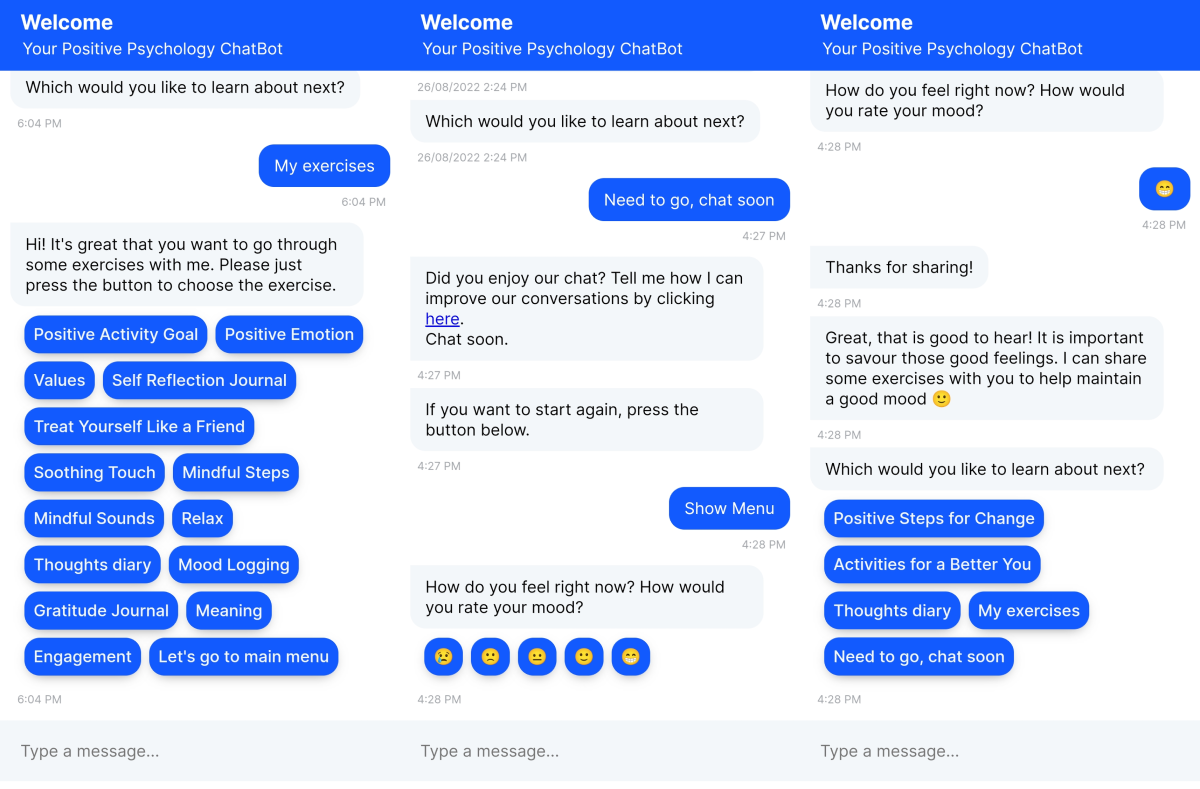
Sample dialogues from the ChatPal chatbot app.

The overall design philosophy used in ChatPal is based on positive psychology, more specifically the PERMA/H model [23,24], which includes 6 core components: positive emotions, engagement, relationships, meaning, accomplishment, and health. ChatPal contains psychoeducational content and exercises such as mindfulness and breathing, mood logging, gratitude, and thought diaries, among other features. All features and content available in the app can be found in Multimedia Appendix 2. Limited amounts of data were available to train the AI in all 4 languages; thus, within the chatbot, users can select from predefined responses (Figure 1). However, ChatPal also has some AI capabilities, so the chatbot will try to understand what the user is asking and direct them to the appropriate conversation. However, if the chatbot does not understand the user input, a fallback message stating the chatbot has limited intelligence will appear and direct users to crisis helplines should they need further help. The full dialogue data sets containing the chatbot utterances in all 4 languages are freely available on the internet [25].

### Recruitment

Participants from rural NPA areas of Europe were recruited to use the chatbot for 12 weeks. Recruitment took place in Northern Ireland (through Ulster University), the Republic of Ireland (through Munster Technological University), Scotland (through third-sector organizations as coordinated by NHS Western Isles), Finland (through the University of Eastern Finland), and Sweden (through Luleå University of Technology and professional networks at Norrbotten Association of Local Municipalities). Eligible participants completed web-based consent forms confirming they met the inclusion criteria for the study as below:

- Lived in a rural area
- Had no previous mental health diagnosis
- Had no previous suicidal thoughts or behaviors in the past year
- Were aged 18 years or older
- Consented to the storage and analysis of anonymous user interactions and other chatbot data

Mental health service users were recruited through Action Mental Health in Northern Ireland. Participants filled out a web-based consent form confirming they met the inclusion criteria. The inclusion criteria for these participants were as follows:

- Lived in a rural area
- Were aged 18 years or older
- Had a history of mild to moderate anxiety or depression
- Were not currently undergoing crisis intervention with medical services at the time of the study or within 12 weeks prior to the study
- Consented to the storage and analysis of anonymous user interactions and other chatbot data

### Study Design

Study participants were recruited over the internet via email to take part in a 12-week single-arm pre-post intervention study with the ChatPal chatbot, as detailed in the previous section. Once eligible participants completed the consent form, they were emailed further instructions on how to download ChatPal to their device and were given a region-specific code to enter during onboarding. Once participants had successfully downloaded the chatbot and entered their regional code, they were automatically assigned an anonymous identifier (a unique ID code for each participant) and were presented with a link to complete the baseline survey. The baseline survey asked for participants’ age, gender, occupation, education level, and outcome measures. The rest of the onboarding experience involved participants consenting to the storage and analysis of their anonymous chatbot usage data. The regional code and anonymous identifier were recorded in all the surveys, so demographic and outcome measures could be linked with user event log data (chatbot usage data). Thus, all data collected were anonymous and linked using participants’ anonymous identifiers.

### Outcome Measures

The primary outcome measure was the Short Warwick-Edinburgh Mental Well-Being Scale (SWEMWBS) [26], and secondary outcome measures included the Satisfaction With Life Scale (SWLS) [27] and the World Health Organization-Five Well-Being Index (WHO-5) [28]. All outcome measures were recorded at baseline, and participants received notifications to complete outcome scales again at the midpoint (week 6) and at the end point of the study (week 12). Reminder emails were sent to participants throughout the 12-week period to encourage the use of ChatPal and the completion of the surveys.

### Statistical Analysis

RStudio (version 3.6.0; RStudio, PBC) and the R programming language were used for data wrangling and statistical analysis. Participant demographics, including gender and age range, were obtained from the chatbot event log data, as these questions were presented to users during onboarding. Participants’ actual age and occupations were ascertained from the baseline survey. SWEMWBS scores were calculated by summing the scores for each of the 7 items and transforming the raw scores into metric scores using the SWEMWBS conversion table [26]. WHO-5 scores were calculated by summing the raw scores and multiplying them by 4 to get a total of 100. SWLS scores were calculated by summing the raw scores to get a total out of 35. Data were assessed for normality using the Shapiro-Wilk test, with *P*<.05 indicating data were not normally distributed. Summary statistics were computed for all outcome measures (SWEMWBS, WHO-5, and SWLS). All outcome measures followed a normal distribution, so parametric tests were applied. A one-way ANOVA was used to compare scale scores from baseline, midpoint, and end point, and *t* tests were used to compare scores between groups, with *P*<.05 considered statistically significant. Chatbot event log data were filtered to only include users that enrolled in the 12-week study, and summary statistics were computed for each user to include the total number of interactions, number of unique days they used the chatbot, and duration of use in days. The log data were then linked with survey data using the anonymous identifier present in both data sets.

### Qualitative Analysis

Feedback in the form of free-text responses to open-ended questions was collected over the 12-week study period. Responses obtained in Swedish and Finnish were translated into English for analysis. This feedback was then analyzed using thematic analysis techniques based on those suggested by Braun and Clarke [29], resulting in a thematic map where the results are reported as frequencies.

## Results

### Participant Demographics

A total of 348 participants took part in the intervention study. The majority of participants were female (n=254, 73%; Table 1). Participants ranged in age from 18 to 73 years, with a mean age of 29.6 (SD 11.7) years, and the most common age group was between 18 and 24 years (n=139, 40%; Table 1). Participants had a wide range of occupations, with the top 5 including students (n=105), teachers (n=16), those that were unemployed (n=11), assistant nurses (n=7), and store employees (n=6).

**Table 1:** Demographic and mental well-being scale scores for participants at baseline.

			Participants
**Age (years), n (%)**			
	18-24	139 (40)	
	25-34	87 (25)	
	35-44	41 (12)	
	45-54	40 (11)	
	55-64	37 (11)	
	>65	4 (1)	
**Gender, n (%)**			
	Male	78 (22)	
	Female	254 (73)	
	Other gender	4 (1)	
	Unknown gender	12 (4)	
**Outcome measures, mean (SD)**			
	Short Warwick-Edinburgh Mental Well-Being Scale (SWEMWBS)	20.43 (3.66)	
	The World Health Organization-Five Well-Being Index (WHO-5)	47.67 (18.22)	
	Satisfaction With Life Scale (SWLS)	20.43 (6.7)	

At the beginning of the study, 83% (n=283) of the total participants completed the baseline survey, while 11.8% (n=41) completed the midpoint survey after 6 weeks, and 6.6% (n=23) completed the end point survey at week 12.

### Changes in Mental Well-Being

A subset of participants (n=98) responded to a feedback survey within the chatbot. Respondents were asked how useful they thought the chatbot was for supporting mental well-being. The majority of participants (n=52, 53%) thought the chatbot was “somewhat useful”, while 18 (18%) thought it was “very useful.” Ten (10%) participants rated it as neutral (neither useful nor useless), 11 (11%) thought it was somewhat useless, and the remaining 7 (7%) thought it was not useful at all. Respondents were also asked how much they believed the chatbot changed their mental well-being, and the majority thought their well-being was slightly improved (n=40, 41%), or unchanged (n=41, 41%), as a result of using ChatPal, while a small number (n=11, 11% thought their well-being significantly improved). Six people reported that using the chatbot affected their mental well-being in a negative way (n=3 slightly worse, n=3 significantly worse). Out of 6 respondents, 5 left written feedback explaining why they thought the chatbot negatively impacted their well-being (Textbox 1).

**Figure 2:**
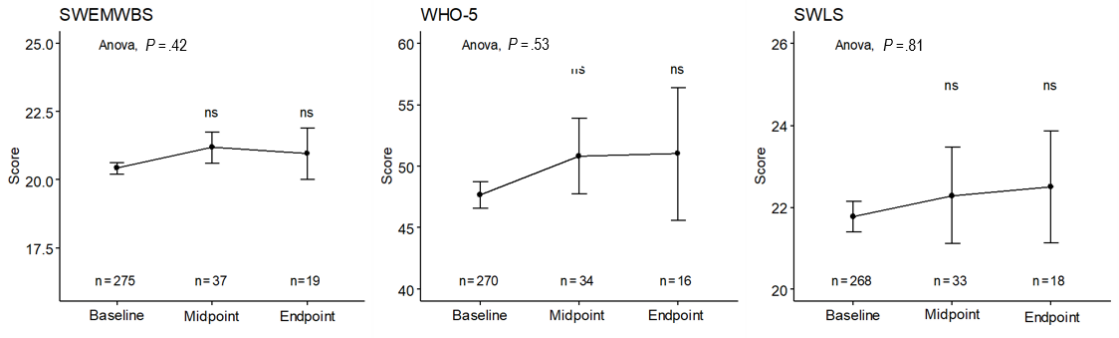
Short Warwick-Edinburgh Mental Well-Being Scale (SWEMWBS), World Health Organization-Five Well-Being Index (WHO-5), and Satisfaction with Life Scale (SWLS) scores across the study period. The mean and standard error for baseline, midpoint, and end point scores, along with significance (mid and end point scores compared to baseline) are shown.

The SWEMWBS was used to measure well-being throughout the study period. Well-being scores improved for individuals from baseline to midpoint and from baseline to end point (Table 2, Figure 2); however, the improvement in scores was not statistically significant (*P*=.42). Across genders, SWEMWBS scores improved nonsignificantly from baseline to midpoint (females: *P*=.99, males: *P*=.18) and from midpoint to end point (females: *P*=.82, males: *P*=.34). SWEMWBS has a mean score of 23.5 and an SD of 3.9 in the general population of the United Kingdom [30]. Participants in this study had an average score of 20.4 at baseline and 21.2 at the end of the ChatPal study (Table 2), indicating well-being scores in this population were lower on average compared to the population norms.

Secondary outcome measures included the World Health Organization-Five Well-Being Index (WHO-5) and SWLS. Participants’ scores on the secondary outcome measures improved throughout the study period; however, the improvements were not statistically significant on WHO-5 (*P*=.52) or SWLS (*P*=.81; Figure 2, Tables 3 and 4). The WHO-5 general population norm for Finland, Sweden, the United Kingdom, and Ireland combined is 63.05 [31], and a cutoff score of ≤50 is typically used for depression [28]. At baseline, participants’ average score was 47.7, but this figure rose to 51.0 by the end of the 12-week period (Table 3). Across genders, WHO-5 scores improved nonsignificantly from baseline to midpoint (females: *P*=.58, males: *P*=.41) and from midpoint to end point (females: *P*=.90, males: *P*=.55). At baseline, SWLS scores averaged 20.4 (Table 4), indicating neutral satisfaction with life, and this score improved to 21.2 (Table 4) at the midpoint, which indicates individuals were “slightly satisfied” with life, according to the benchmark scoring for this scale [32].

**Figure 3:**
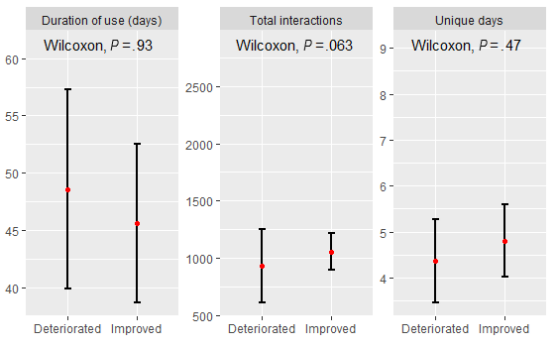
Usage statistics for those who improved in Short Warwick-Edinburgh Mental Well-Being Scale (SWEMBS) from baseline to midpoint. Mean (red) and standard error shown.

A higher number of participants completed the primary outcome measure (SWEMWBS) at baseline and midpoint (n=37) compared to those who completed all 3 baseline, midpoint, and end point surveys (n=12). Of the 37 that completed the first 2 surveys, 16 (43%) individuals had higher SWEMWBS scores at the midpoint, indicating improved well-being (mean change 2.34, SD 2.24), 19 (51%) individuals had lower SWEMWBS scores at the midpoint, indicative of declining mental well-being from baseline to the midpoint (mean change 2.43, SD 1.77), and the remaining 2 (6%) had no change in scores. The group that displayed improved mental well-being from baseline to midpoint was significantly younger (mean age 27 years) compared to those who had worse mental well-being (mean age 37 years; *P*=.03); however, there were no significant differences in gender between the 2 groups (*χ*^2^_1_=0.92, *P*=.34). The group that had improved mental well-being typically had a shorter duration of use with the chatbot, but during this time they interacted with the chatbot on more unique days and logged a higher number of interactions than the group that had decreased mental well-being (Figure 3).

**Feedback from Swedish and Finnish participants who responded to a survey stating the chatbot made their well-being slightly worse or significantly worse.**

- “Det går väldigt segt och uppfyller inte mina förväntningar varken i användarvänlighet eller vad de olika delarna handlar om.” (It’s very slow and doesn’t meet my expectations, neither when it comes to user-friendliness nor what the different sections are about.)
- “Ei ymmärtänyt mistä haluan puhua ja kun kysyin kysymyksen, alkoi tarjota hyödyttömiä ohjeita ihan randomisti.” (Didn’t understand what I wanted to talk about and when I asked the question, started offering useless instructions just randomly.)
- “Nyt alkoi kiukustuttaa kun päästäkseni alkuvalikkoon meni kohtuuttoman kauan aikaa… Chatpal vastasi todella hitaasti, serveri ei toiminut.” (Now I was beginning to feel annoyed because it took an unreasonably long time to get to the main menu… Chatpal responded very slowly, the server did not work.)
- “Chatpalin kommentteja ei jaksa odottaa, on aivan liian hidas. Nyt tuli paljon virheitä. En saanut tehtyä haluamaani harjoitetta kohtuullisessa ajassa virheiden vuoksi.” (I’m tired of waiting for Chatpal’s comments, it’s way too slow. There were lots of mistakes this time. I was not able to finish the exercise I wanted within a reasonable time.)
- “Chat palin käyttöön ei ole ollut nyt motivaatiota vaikka ymmärrän, että sen tehtävänä olisi juurikin auttaa jaksamaan. Ohjelman tekniset ongelmat haastavat eniten käytön aloituskynnystäni. Jos toimisi sujuvasti, olisin käyttänyt tod näk useammin ihan ‘periaatteesta” (I haven’t had motivation to use Chatpal lately, even though I understand that the aim of the chatbot is to help me to carry on. Technical issues raise the most my threshold to start using it. If it worked smoothly, I probably would have used it more often as a matter of principle.)

**Table 2:** Short Warwick-Edinburgh Mental Well-Being Scale (SWEMWBS) scores for participants throughout the 12-week study period.

	Range	Mean (SD)	Median (IQR)	High well-being, n (%)^a^	Low well-being, n (%)^b^
Baseline (n=275)	9.51-35.00	20.43 (3.66)	19.98 (4.65)	15 (5.45)	117 (42.55)
Midpoint (n=37)	15.32-29.31	21.19 (3.68)	20.73 (5.52)	3 (8.11)	18 (48.64)
End point (n=19)	12.40-29.31	20.95 (4.50)	21.54 (6.04)	1 (5.26)	8 (42.11)

^a^Top 15% population scores (27.5-35).

^b^Bottom 15% population scores (7.0-19.5).

**Table 3:** The World Health Organization-Five Well-Being Index (WHO-5) scores for participants throughout the 12-week study period.

	Range	Mean (SD)	Median (IQR)	High well-being, n (%)^a^	Low well-being, n (%)^b^
Baseline (n=270)	4-88	47.67 (18.22)	48.00 (24.00)	115 (43)	155 (57)
Midpoint (n=34)	12-84	50.82 (18.01)	48.00 (20.00)	16 (47)	18 (53)
End point (n=16)	12-76	51.00 (21.54)	60.00 (26.00)	11 (69)	5 (31)

^a^Score of ≥50.

^b^Score of <50.

**Table 4:** Satisfaction With Life Scale (SWLS) scores for participants throughout the 12-week study period.

	Range	Mean (SD)	Median (IQR)
Baseline (n=268)	9.51-35	20.43 (6.7)	19.98 (4.65)
Midpoint (n=33)	15.32-29.31	21.19 (3.7)	20.73 (5.52)
End point (n=18)	12.40-29.31	20.95 (4.5)	21.54 (6.04)

### Qualitative Analysis of User Feedback

**Figure 4:**
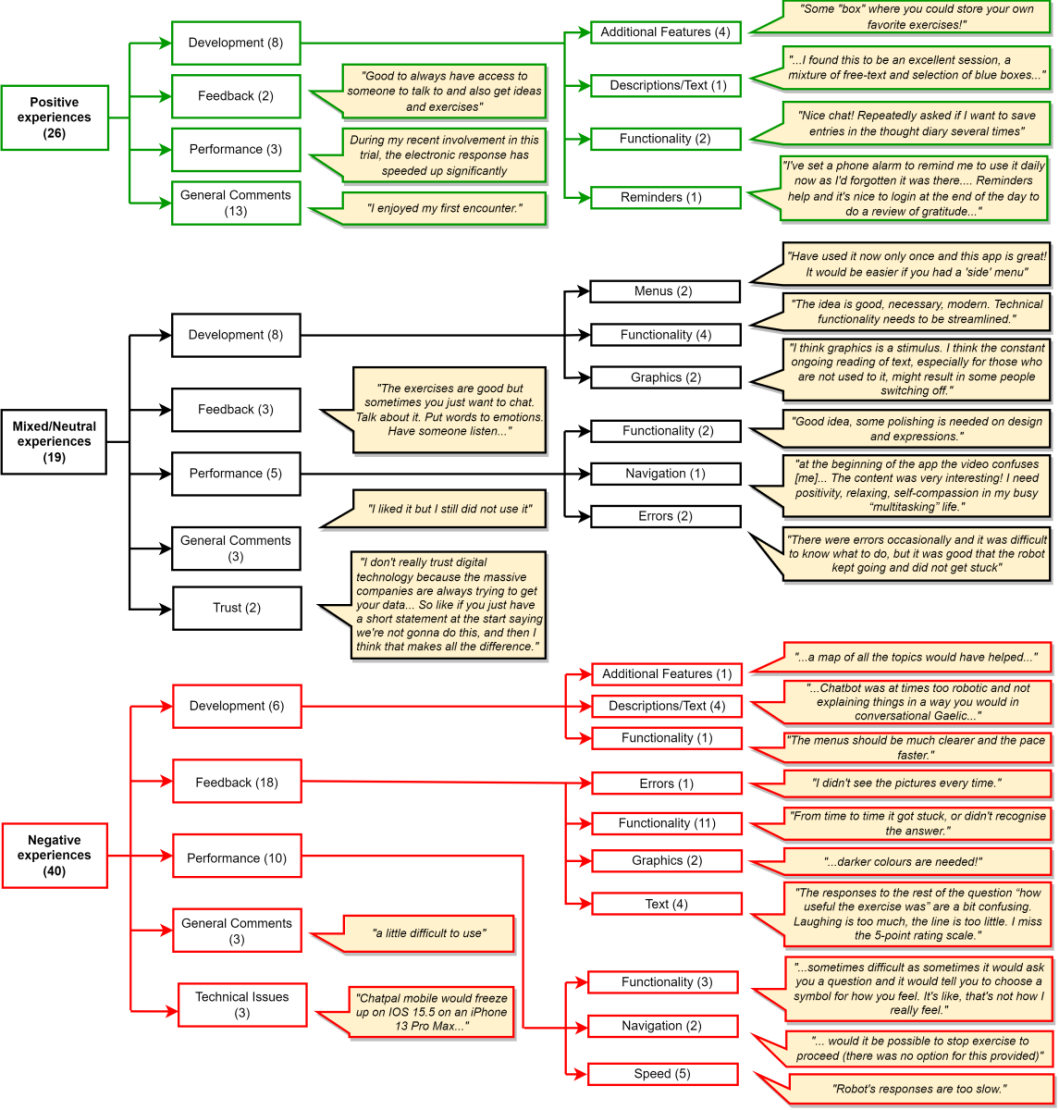
Themes identified from ChatPal feedback, with examples of user comments for each subtheme.

Thematic analysis was conducted based on written free-text feedback (n=87) from participants. Three main themes were identified: “Positive experiences,” “Mixed or neutral experiences,” and “Negative experiences” (Figure 4). Across these main themes, 4 common subthemes emerged, including “development,” “feedback,” “performance,” and “general comments,” with an additional subtheme “technical issues,” which was specific to “negative experiences,” and “trust,” which was linked to “mixed or neutral experiences.”

When discussing positive aspects around feedback and general comments, participants commented that they liked the different exercises offered in the chatbot and activities such as the gratitude diary, which prompted feelings of positivity (Figure 4). Some individuals who left positive comments gave suggestions for future development, for example, a place to store favorite exercises and be able to set reminders to use the app (Figure 4).

Some participants had a mixed or overall neutral experience with the chatbot, with 1 person stating that they liked it but still did not want to use it, and another commenting that they liked the content but still wanted to discuss their feelings with a human (Figure 4). Most of the comments with mixed or neutral aspects mentioned that they liked the chatbot overall but that there were some barriers, such as technical errors or confusion around navigating content, that needed to be overcome. Specific comments left by 2 participants stated that they would like a clearer, easy-to-use menu, and several other participants noted menu navigation as something that could be improved (Figure 4). Trust was mentioned, and participants stated that they would be more likely to trust the chatbot if it was transparent about its purpose and what would happen to user data at the very start (Figure 4).

A few neutral and negative experiences were noted, specific to the Scottish Gaelic version of the app. First, the native Scottish Gaelic speakers who took part were not accustomed to seeing the language written down, as typically the language is spoken more often than it is read. Second, for users reading Scottish Gaelic translations, some of the language around mental health and well-being were not common terminology in day-to-day conversations, and several participants thought that having a Gaelic glossary would be helpful.

Negative experiences mostly included technical issues with the chatbot’s functionality (Figure 4). At the beginning of the study, participants commented on the app’s performance, stating that it was slow to respond. This information was shared with the developers so that improvements could be made while the study was ongoing, and later feedback from participants mentioned this in a positive manner, noting the chatbot responses were considerably faster (Figure 4). A few participants disliked the choice of 5 different icons (emojis) to rate their mood from happy to sad, as they thought this range was not wide enough to express how they felt at the time (Figure 4).

## Discussion

### Principal Results

This study reports on the evaluation of a multilingual chatbot named ChatPal, designed as a mental health promotion tool to promote positive mental well-being in individuals across sparsely populated areas of Europe. A total of 348 participants were recruited to use the chatbot for a 12-week period, with outcome measures recorded at the beginning, after 6 weeks (midpoint), and after 12 weeks (end point). This study benefited from the inclusion of participants with a wide range of demographics, including adults from the ages of 18 to 65 years or older. In response to the survey question, which asked participants how they believed the chatbot improved their mental well-being, over half (51/98, 52%) said that it significantly or slightly improved. Only a few participants (n=6) thought the chatbot negatively impacted their well-being, and the reasons for this were due to technical errors or frustration at the slow speed of chatbot responses, which was later rectified. This is an important point for trials of digital interventions to address; that is, any technical issues can be an unwanted confounder and negatively affect the outcome variables. Hence, it is crucial that technical testing be rigorous and that studies incorporate a pilot to address technical issues or barriers ahead of the main intervention study or trial. The outcome measures that were recorded at baseline, midpoint, and end point revealed that participants’ well-being generally improved throughout the study period; however, the improvements in well-being were not statistically significant for SWEMWBS, WHO-5, and SWLS. Nonsignificant improvements in well-being were similar for both males and females. The SWEMWBS scores at baseline and throughout the 12-week period remained below the population norms for the United Kingdom [30]. However, by the end of the trial, WHO-5 scores improved to 51, which was just above the threshold for depression (a score of 50 or below) compared to the scores at baseline, which were below this threshold.

It is important to characterize users who showed improvements in well-being after using ChatPal, as even if it only helps a small proportion of users, it could be seen as beneficial. Individuals that did show improvements in well-being (n=16) interacted more with the chatbot and were significantly younger on average (age 27 years) compared to those whose well-being worsened over the study, with an average age of 37 years. This may be related to the likely acceptance and adoption of SMS text messaging and chatbots among younger generations, who are also considered to be the “generation mute.” Generation mute is the nickname given to people younger than 25 years, who have grown up in the digital era and predominantly use smartphones for messaging as opposed to voice calls. It may be the case that those who are younger are more familiar with text-based apps’ benefit more than those who are older.

Given that the results showed marginal improvements in mental well-being in those who used ChatPal, the app could be thought of as just 1 tool of many in a larger service offering that could be used in conjunction with other digital or face-to-face services. There is potential for ChatPal to be used with those who are on waiting lists for mental health services or outpatients who have already received counseling, for example. ChatPal could be thought of as a health promotion tool that can be used by anyone in the general population, providing means of learning and education about what it means to be in a good state of mental well-being and as a preventative tool to stop mental health problems from escalating. It can also provide information about mental health, signs of mental illness, and tips about giving support, which can improve mental health literacy in the population and increase public acceptance of mental health care [33]. Educating people about mental health may help to minimize stigma and increase confidence in taking appropriate action [3]. Participants stated they were somewhat unfamiliar with mental health terminology in Scottish Gaelic; this further supports the use of ChatPal or other mental health promotion apps as educational tools that could be used to promote well-being in different languages.

Some participants mentioned that they would prefer to open up to a human regarding their feelings. Others may be more likely to open up to a chatbot, as it allows them to remain anonymous. Previous studies have shown patients disclose more sensitive information to a chatbot when compared to a human therapist [34] and that people sometimes engage more on a personal level with web-based therapists [16].

Ultimately, a personalized approach to mental health and well-being would be beneficial, as what works best for each person needs to be considered on an individual basis, and some may prefer to use multiple different tools to look after their mental well-being.

### Comparison With Prior Work

This paper further supports the need for blended service offerings, given that mental well-being scores did not significantly increase. This result is in line with findings from recent reviews [7,35], which found that blended service offerings were more effective in treating those living with depression, and several studies have shown that guided interventions resulted in better adherence and lower dropouts compared with unguided interventions [36-38].

Previous studies [14,15] used chatbots with a decision tree structure and prewritten text, which is similar to the ChatPal chatbot. Almost all other mental health chatbots, for example, Woebot [4] and Wysa [13], are targeted at treating mental illness using cognitive behavioral therapy and dialectical behavior therapy, unlike ChatPal, which focuses more on mental health promotion. ChatPal is built around the PERMA/H positive psychology framework [23,24] and includes evidence-based practices. The ChatPal chatbot is also available in 4 languages, including English, Swedish, Finnish, and Scottish Gaelic, which is unique. Previously, AI mental health chatbots have occasionally generated inappropriate responses [39], which can be problematic if the user is disclosing concerning information about their mental health. ChatPal contains limited AI, and if it does not understand user input, it will signpost the user to crisis helplines should the user need additional help.

A recent paper by Torous et al [40] highlighted the future importance of accelerated use of digital mental health tools but also pointed out that there are barriers to overcome, such as training staff, high-quality evidence, and digital equity. In this study, only a small subset of users who started the study actually completed it. Adherence is a challenge with digital health intervention studies in general, and high drop-off is typical with mental health apps. A previous study looked at real-world data on health app usage and found that, on average, only 3.9% of users continued to use these apps after 15 days [41]. In the future, it will be important to find strategies that will increase engagement and adherence in mental health app trials.

### Limitations

This study included a wide age range of participants; however, the majority of participants were female (254/348, 73%). The chatbot study took place across 4 European countries, but a high proportion of participants were from Sweden. As the study design was a single-arm pre-post study, another limitation is the lack of a randomized controlled environment and control group, which has the potential to cause bias in the study and limits the findings regarding the chatbot’s causality on mental well-being. In addition, no power calculation was conducted a priori. Given the small number of participants that completed the outcome measures at the end point (n=19) and that we did not find any significant changes in well-being scores, this could be the result of small sample size and underpowering. The investigators did not select specific individuals for inclusion in the study; however, as participants self-selected to take part in the study based on the inclusion criteria, it is possible that self-selection bias may have been introduced into the study. Participant adherence in the study may have been better if investigators were able to interact with participants face-to-face; however, most interactions and engagement were carried out over the internet due to the pandemic. However, this study could be considered somewhat “real world” given the limited interaction participants had with researchers (eg, consent was taken electronically). The participant feedback obtained was anonymous and could not be linked to the survey data, so we were unable to look at specific feedback across age groups, gender, and those who exhibited changes in well-being.

### Conclusions

The ChatPal chatbot had a marginal effect on mental well-being, although this was not statistically significant. Interestingly, the majority of users believed that the chatbot slightly or significantly improved their mental well-being. Those who had improved mental well-being had more interactions with the chatbot and were younger in age. Future work could also include analyzing feedback from chatbot users for specific age groups, as requirements for mental health chatbots and digital technologies, in general, should be considered across different generations. The ChatPal chatbot can be seen as a mental health promotion tool that targets the general population rather than treating those with mental illness. The results of this study support this, and we propose that ChatPal could be included as part of a blended service that complements other digital tools as well as face-to-face service offerings. Further research is needed to confirm the efficacy of ChatPal alongside other complementary services.

## Appendix

Multimedia Appendix 1 ChatPal chatbot architecture.

Multimedia Appendix 2 Overview of ChatPal chatbot dialogues and features.

## Abbreviations

AI: artificial intelligence
NPA: Northern Periphery and Arctic
SWEMWBS: Short Warwick-Edinburgh Mental Well-Being Scale
SWLS: Satisfaction With Life Scale
WHO-5: World Health Organization-Five Well-Being Index

## Data Availability

The ChatPal chatbot dialogue data, which contains the chatbot utterances in English, Swedish, Finnish, and Scottish Gaelic, are freely available from Ulster University and can be accessed from [25]. The survey and event log data sets generated during and analyzed during this study are not publicly available as participants consent included data sharing within the project research team only.
