# Supplementary figures and images for: A Multilingual Digital Mental Health and Well-Being Chatbot (ChatPal): Pre-Post Multicenter Intervention Study
Source: J Med Internet Res. 2023 Jul 6;25:e43051. doi: 10.2196/43051 (PMC10359914; doi:10.2196/43051)

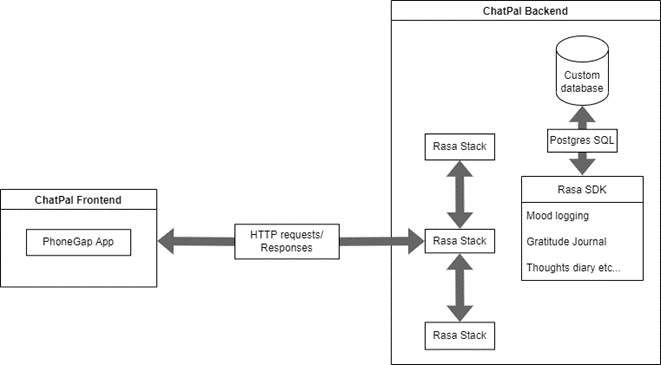

Supplement: Multimedia Appendix 1 [file jmir_v25i1e43051_app1.png]

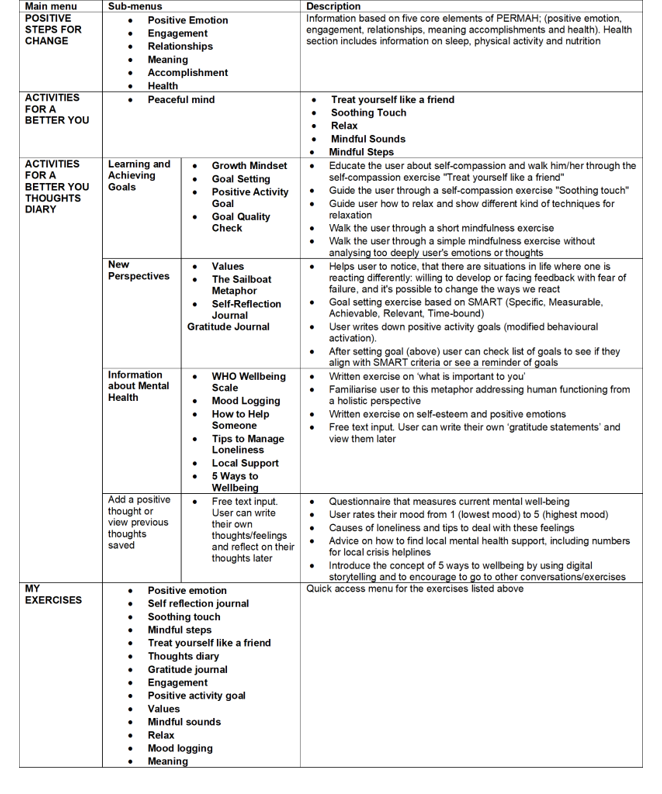

Supplement: Multimedia Appendix 2 [file jmir_v25i1e43051_app2.png]
